# Rater agreement for assessment of equine back mobility at walk and trot compared to quantitative gait analysis

**DOI:** 10.1371/journal.pone.0252536

**Published:** 2021-06-04

**Authors:** T. J. P. Spoormakers, E. A. M. Graat, F. M. Serra Bragança, P. R. van Weeren, H. Brommer

**Affiliations:** 1 Department of Clinical Sciences, Equine Division, Faculty of Veterinary Medicine, Utrecht University, Utrecht, The Netherlands; 2 Department of Animal Sciences, Adaptation Physiology, Wageningen University and Research, Wageningen, The Netherlands; University of Lincoln, UNITED KINGDOM

## Abstract

**Background:**

Lameness assessment in horses is still predominantly performed using subjective methods. Visual assessment is known to have moderate to good intra-rater agreement but relatively poor inter-rater agreement. Little is known about inter- and intra-rater agreement on the evaluation of back motion, for which no objective measurement technique in a clinical setting is available thus far.

**Objectives:**

To describe inter- and intra-rater agreement of visual evaluation of equine back mobility.

**Study design:**

Rater reliability study using a fully crossed design in which all horses are rated by all observers. This data is compared with objective gait analysis.

**Methods:**

Seventy equine professionals (veterinarians and physiotherapists) and veterinary students evaluated videos of 12 healthy horses at walk and trot on a hard, straight line. Nine parameters related to back mobility were scored: general mobility, thoracic, lumbar, lumbosacral flexion and extension and left and right thoracolumbar latero-flexion. All parameters were compared with simultaneously measured quantitative motion parameters. After 1 month, six randomly chosen horses were re-evaluated by 57 observers.

**Results:**

For each parameter inter- and intra-rater agreements were calculated using intra-class correlation coefficients. For all parameters, inter-rater agreement was very poor (<0.2). The mean intra-rater agreement of all observers and for all parameters was poor (~0.4) but varied between 0.0 and 0.96 for individual observers. There was no correlation between the visual subjective scoring and objective gait analysis measurements.

**Main limitations:**

Horses were scored from videos and by lack of any existing (semi-) quantitative system, a custom-made system had to be used.

**Conclusions:**

The poor inter- and intra-rater agreements of visual scoring of mobility of the equine back and the disagreement between subjective and objective gait analysis data, demonstrate the need for the development and introduction of objective, quantitative and repeatable techniques to assess equine back motion.

## Introduction

The equine back is generally acknowledged to be a pivotal element of the horse’s musculoskeletal system of which malfunctioning is a common cause of poor performance. Therefore, clinical examination of back function is a critical skill of the equine practitioner in clinical cases of lameness or poor performance, and during prepurchase exams in presumably healthy horses. In the past decades, little attention has been given to the clinical examination of the back for which no real standard exists, and which is still performed differently between and within groups of professionals, such as veterinarians and physiotherapists. This is in contrast with the attention given to observational and experimental studies focusing on a better understanding of the biomechanics of the equine thoracolumbar spine [1–6].

The clinical examination commonly starts with a visual assessment of the mobility during a dynamic exam during which back motion is judged to be normal, increased or decreased, where the latter two categories could point at the possible existence of a back problem.

It has been proven that visual assessment of equine gait, especially in hind limb lameness, is difficult and highly subjective with a low inter-rater reliability [7–9]. The same may be true for visual assessment of mobility of the equine back, but data on inter- and intra-rater reliability are lacking. The aim of the present study was to evaluate the level of agreement of visual assessment and scoring of mobility of the equine back between different observers and compare this with data from quantitative analysis of spine motion.

The hypotheses were that: 1) fair to good inter-rater agreement would exist between experienced observers, versus poorer inter-rater agreement between less experienced observers; 2) intra-rater agreement would be fair to good for both experienced and less experienced observers; 3) there would be a good correlation between the objectively measured quantitative parameters and the visual, subjective scoring of back motion by experienced observers, but correlation would be much less with scoring by less experienced observers.

## Material and methods

Data collection took place in the Netherlands. According to Dutch legislation and regulations, ethical approval is not required for non-invasive experiments where animals are not subjected to any additional risks related to the study, outside normal handling. Thus, no ethical permission was required for this study. All horses were in possession of the Faculty of Veterinary Medicine, Utrecht University, the Netherlands, obviating the need for consent by private owners. All participants were contacted with the following statement: “*You are invited to participate in a survey for the evaluation of equine back motion*. *The purpose of the survey is to gain understanding in rater agreement of visual evaluation of the equine back”*. Only after confirming by email their willingness to participate, invited participants were allowed to enter and start the survey. Input from the participants was anonymized before processing for the determination of intra -and inter-rater agreement and comparison with objective measurements.

### Horses

Healthy, riding horses, that were used daily for education, student riding, and driving purposes, and that were deemed sound at clinical examination were enrolled in this study. All horses were kept at the Faculty of Veterinary Medicine, Utrecht University, the Netherlands. All horses were equipped with 12 mm soft spherical reflective markers (Qualisys AB^a^) using double-sided adhesive tape. Three markers, as a cluster, were mounted on the frontal plane of the head; to the front and hind legs brushing boots with clusters of respectively 3 and 4 makers were attached. The axial skeleton was marked with markers on the highest point of the withers, on top of the dorsal spinal processes (DSP) of T12, T15, T18, L3, L5 and S5, and between both tuber sacrale (TS). Additional markers were attached at the proximal point of the spina scapula, both tuber coxae and 15 cm paramedian to the left and right side of the DSP of T12, T15, and T18. More additional markers were placed on the last rib, and at the point of widest excursion of the ribcage of T12, and T15. These additional makers were not used in this study. The position was determined by palpation by the same researcher (FSB) for all horses.

### Kinematic data analysis

All kinematic data was filtered, and analysed as previously described [10]. Segment angles (T12, T15, T18, L3, L5, S5, TS) were calculated using the markers cranial and caudal to the marked vertebra in question. Range of motion of the whole back was calculated using two segments ‘withers-T15’ and ‘T15- TS’, in the sagittal plane for flexion extension (FE) and in the horizontal plane for lateral bending (LB). Pelvic roll (axial rotation), pitch (FE) and yaw (LB) were calculated as projection angles in the frontal, sagittal and dorsal planes, respectively using the markers at TS and both tuber coxae. The back data was processed by previously described methods using Matlab 2019b^b^ [10].

### Video and data collection

**Fig 1:**
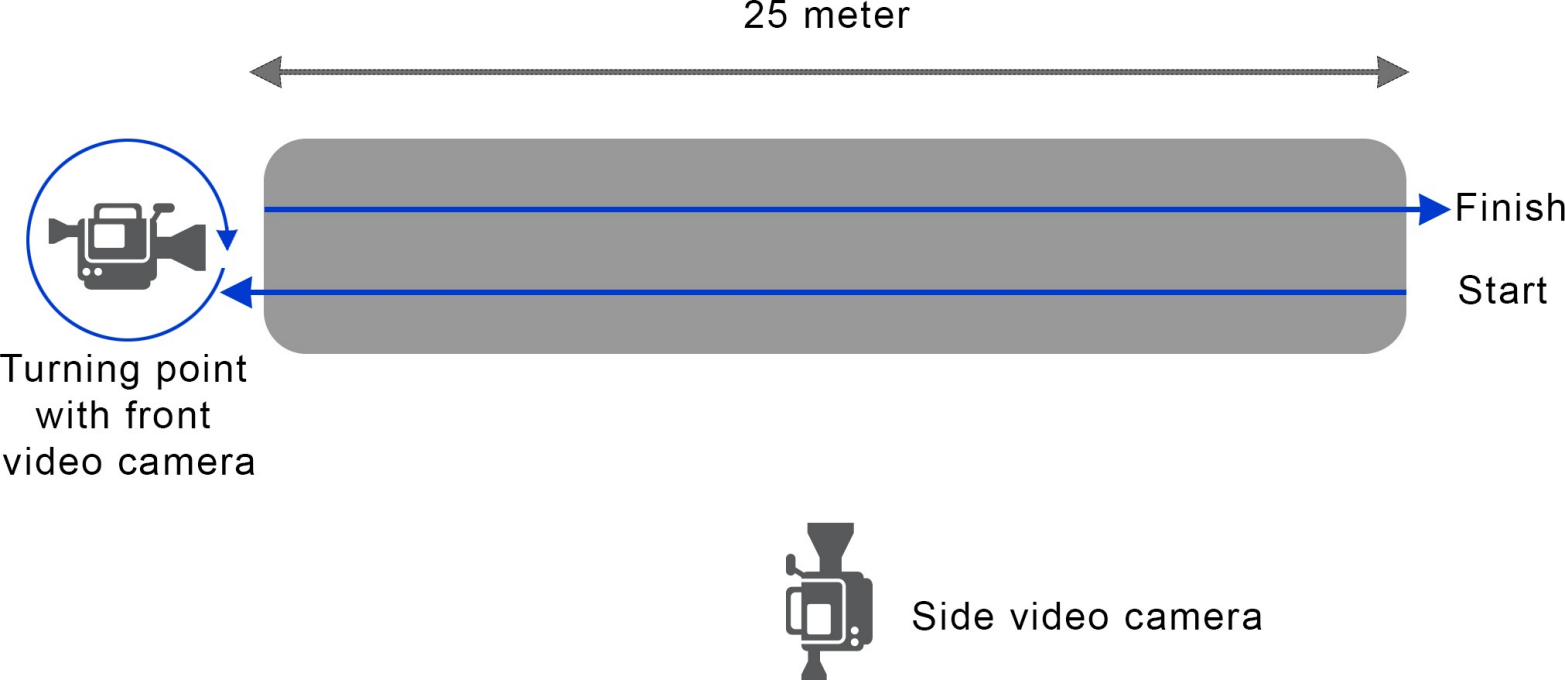
Setup of experiment: 25 meters hard straight line, showing start, finish and the position of front and side video cameras used to film the horses during walk and trot, back and forth.

Frontal and lateral videos of each horse were simultaneously made during walk and trot on a 25 meters straight indoor track with a hard surface (Fig 1). These recordings resulted in 48 videos (frontal and lateral videos from 12 horses in walk and trot; 4 videos/ horse). All horses were presented by the same handler and the lateral videos were recorded from the left side. Simultaneously with these video recordings optical motion capture data were recorded using Qualisys Track Manager software^a^ (QTM version: 2.17 built 4000) connected to 18 high-speed infrared cameras (Oqus 700+) set at a sampling frequency of 200 Hz.

### Observers

A total of 460 Dutch equine veterinarians, 101 equine physiotherapists and 84 veterinary students (master track equine medicine) were invited to participate in an online survey. The observers were requested to specify their profession: equine physiotherapist, board-certified equine specialist, equine practitioner, general/mixed veterinary practitioner, or student, and additional certificates as chiropractor (Focus on Equine Spine, International Veterinary Chiropractic Association, Backbone Academy). The observers were also requested to specify whether they worked part- or full-time, and in which year they graduated. Experienced observers were defined as having more than 5 years of experience.

### Survey

**Fig 2:**
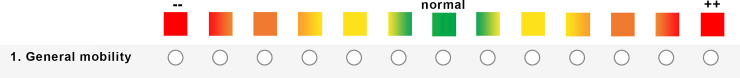
Example of a coloured bar with tick boxes for evaluating general mobility of the back (for analysis the score was given a value of 1 to 13. e.g., the left tick box was labelled with 1 (no movement (—)), the middle with 7 (normal mobility), and the right one (severe hypermobility (++)) with 13).

**Fig 3:**
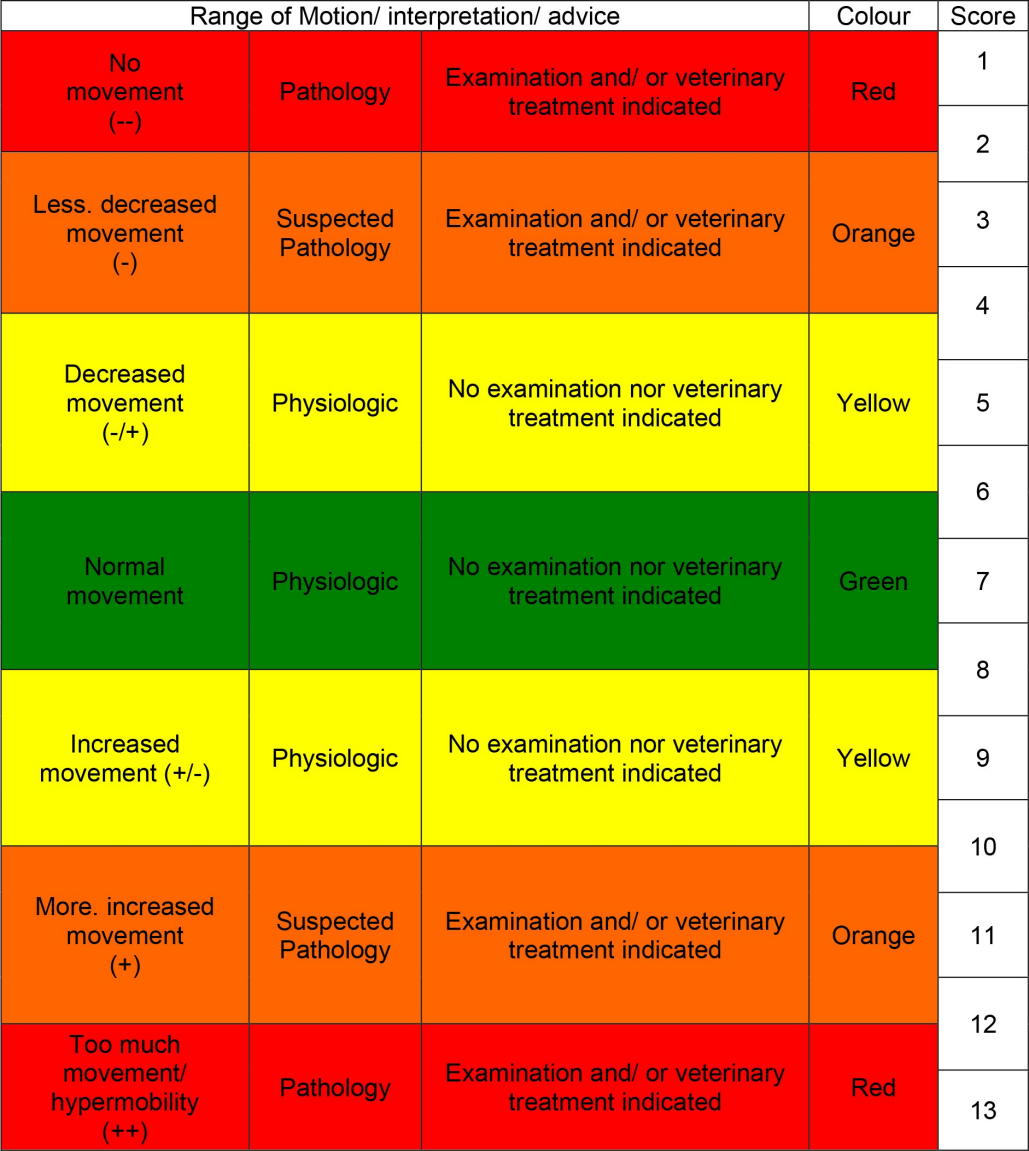
Colour scheme used to grade back mobility on a 1–13 scale associated with range of motion. The interpretation of the grades is given in terms of existence of pathology and in clinical terms with respect to the need to take action.

An on-line Survey Monkey was created and sent to the observers asking them to evaluate the mobility of the equine back in walk and trot using the video recordings. The survey consisted of two parts. Both parts started with a short introduction explaining the purpose of the study and the scoring system, followed by three examples. Mobility scoring was performed by each observer through ticking one box in a set of 13, coded with different colours (Fig 2). The colours represented various degrees of mobility that were coupled to an interpretation about the existence of pathology and a clinical conclusion with respect to the need to take action (Fig 3). Apart from the “normal/ physiologic/ green” score (7), all other scores indicated altered mobility. This was either decreased (towards the left side of the scale) or increased (towards the right side of the scale). The colour yellow (scores 5 or 9) had to be used when the person scoring deemed the mobility was still within the physiological range. The colours orange and red (scores 1, 3 and 11, 13) were meant to be used when the change in mobility was deemed beyond the physiological range. Box 4 or 10 could be ticked when the observer was in doubt whether the mobility was physiological or as aphysiological, 3 and 11 could be ticked when the observer rated the mobility as slight aphysiological, 2 and 12 and moderate aphysiological and 1 and 13 as severe aphysiological (S1 Fig). Only one box per parameter could be ticked. Nine parameters of back mobility were evaluated: general mobility (GenMob), thoracic flexion (ThorFlex), thoracic extension (ThorExt), lumbar flexion (LumbFlex), lumbar extension (LumbExt), lumbosacral flexion (LumbSacFlex), lumbosacral extension (LumbSacExt), left thoracolumbar latero-flexion (LLatThorFlex), and right thoracolumbar latero-flexion (RLatThorFlex).

The frontal and lateral videos of each horse at each gait (walk or trot) were time synchronised, combined in one screen, and displayed simultaneously. This resulted in 24 (12 horses x 2 gaits) evaluations for the first part of the survey. The survey could be stopped and restarted at any moment but should be finished within 1.5 months after starting the survey. Observers could look as many times at the videos of one horse, as they wanted. However, before evaluating the next horse an evaluation should be completed (all nine parameters ticked) and submitted. A completed evaluation could not be changed, and only completely finished surveys were used for data analysis. Horses and gaits were presented randomly, but in the same order for each participant. The observers did not have prior knowledge about the health status of the horses. One month after closure of the first part, observers who completed this first part of the survey, were invited again for the second part. Of the initial 12 horses, a randomised set of 6 horses, 3 in walk and 3 in trot, had to be evaluated in the same way as in the first part using the same videos.

### Data analysis

Statistical analysis was performed in SAS 9.4^c^. Descriptive statistics (mean, standard deviation (SD)) were calculated for each of the nine parameters that were scored by each observer and for both sessions. To study the relation between the scores on the different parameters given by the same observer, Pearson Correlation Coefficients were calculated. As data for each parameter had a normal distribution, Intraclass Correlation Coefficients (ICCs) were used to assess inter- and intra-rater agreement. Model residuals were checked for normality by visually examining the studentized residuals plots and checking whether skewness and kurtosis were between -2 and 2. For all models, a normal distribution was seen.

For each parameter and gait, the inter-rater reliability was estimated with a generalised linear regression model (PROC MIXED. SAS 9.4^c^) with two-way random effects based on single measurement and absolute agreement (ICC (2.1) [11, 12]. Horses and observers were considered to be a random sample out of a large population of observers and horses. An ICC below 0.5 indicates poor reliability and thus poor inter-rater reliability, between 0.5 and 0.75 moderate reliability, between 0.75 and 0.9 good, and above 0.9 excellent [12]. First, the ICCs were calculated for all the parameters with an intercept-only model. To check whether education, expertise or speciality of each observer affected the ICC, the following variables were put into the model as fixed effect one by one to test their effect on the scoring of the 9 aspects in the two gaits: Working with equine patients (‘100%’ for raters that work with equine patients fulltime and ‘<100%’ for raters that work with equine patients less than fulltime), Student Master track Equine (‘yes’ and ‘no’), Certified Equine Veterinarian (‘yes’ and ‘no’), Experience (‘Inexperienced Student’, ‘Inexperienced Veterinarian’, ‘Experienced Veterinarian’, ‘Inexperienced Physiotherapist’ and ‘Experienced Physiotherapist’, where experienced means >5 years’ experience and inexperienced <5 years’ experience, Chiropractor (FES, IVCA, Backbone Academy) (‘yes’ and ‘no’), European Specialist Equine Internal Medicine (dipl. ECEIM)) (‘yes’ and ‘no’), European Specialist Equine Surgery (dipl. ECVS) (‘yes’ and ‘no’), European Specialist Equine Veterinary Sports Medicine (dipl. ECVSMR) (‘yes’ and ‘no’), General Practitioner (‘yes’ and ‘no’), ISELP certified (‘yes’ and ‘no’), Pre-purchase Equine Veterinarian (‘yes’ and ‘no’) and Resident (veterinary medicine) (‘yes’ and ‘no’).

For each of the 57 observers that repeated the evaluation, the intra-rater reliability was estimated with a generalised linear regression model (PROC MIXED. SAS 9.4^c^) with one way ANOVA and random effect of horse based on absolute agreement (ICC (1.1)) [11]. The analysis resulted in 57 intra-class correlation coefficients (ICC) for each parameter. Per parameter, the 57 ICCs were averaged, and standard deviation, median and interquartile range were calculated and presented in box-whisker plots. Furthermore, linear regression was performed on the ICCs (intra-rater reliability) to test for differences in the expertise of the observers.

Comparison between visual scoring of the observers and objective gait analysis measurements was done by calculating Pearson Correlation Coefficients for each observer score with the objective score, resulting in 70 records for each of the parameters of back mobility. This was performed separately for walk and trot. Data distribution (mean, SD, minimum and maximum) were calculated. Because data was normally distributed, and thereby mean and median values were almost identical, only the mean values are presented.

## Results

### Horses

Twelve horses (10 Dutch Warmblood (KWPN) and 2 Friesian horses; 11 mares and 1 gelding) were used in this study. All were sound at clinical examination. The mean age was 13 years (range 5–22 years), mean withers height was 163 cm (range 155–168 cm), and mean body mass was 608 kg (range 519–712 kg).

### Observers

In total, 135 of the invited persons (645) agreed to participate, and 10.9% (70/ 645) completed the first part of the survey. Fifty-seven of these also completed the second part. The 70 observers included 48 veterinarians, 12 equine physiotherapists and 10 veterinary students. Their qualifications are listed in Table 1. Twelve observers had a certificate of chiropractor of which eight had a veterinary degree and the other four were equine physiotherapists. These physiotherapists all had a degree in human physiotherapy and additionally a certificate in animal physiotherapy. All participants were divided according to the most distinctive degree in education, and thereby prevailed degree above certificate. In cases, no distinction could be made for veterinarians, the participant was labelled as a general practitioner.

**Table 1:** Descriptive statistics of invited participants and those that eventually completed the first and second part of the survey.

Participants Survey							
Group	Invited	Started (%)	Qualifications	Completed first (%)	%	Completed second (%)	%
Physiotherapists	101	22 (21.8)	Equine Physiotherapist	12	11.9	12	11.9
Veterinarians	460	92 (20.0)	Certified Dutch Equine Veterinarian	28 (6.0)	10.4	18 (3.9)	7.8
			Dutch Equine Prepurchase Veterinarian	7 (1.5)		7 (1.5)	
			Equine Specialist Internal Medicine/ Surgery or Veterinary Sports Medicine and Rehabilitation Diplomate ECEIM^†^/ ECVS^‡^/ ECVSMR^§^)	6 (1.3)		6 (1.3)	
			General Practitioner	6 (1.3)		4 (0.8)	
			Resident (ECVS^‡^)	1 (0.2)		1 (0.2)	
Students	84	21 (25.0)	Veterinary Students (Equine Master Track)	10	11.9	9	10.7
Total	645	135 (20.9)		70	10.9	57	8.8

^†^ European College of Equine Internal Medicine

^‡^ European College of Veterinary Surgeons

^§^ European College of Veterinary Sports Medicine and Rehabilitation.

### Inter-rater reliability

Of the total 15,120 scores (70 observers x 12 horses x 2 gaits x 9 parameters) 72.0% (10,886/15,120) were below 7 (green), indicating decreased movement. In 34.7% (5,250/15,120) these scores were below 5 and in 2.4% (370/15,120) above 9, implying that the observer deemed further examination and/or treatment necessary (Fig 3). The mean scores per parameter at walk ranged between 5.29 (SD: 2.02) for LumbSacFlex and 6.10 (SD: 2.11) for LLatThorFlex, respectively (S1 Table). In trot, mean scores were lower and ranged between 4.78 (SD: 1.87) for LumbSacFlex and 5.24 (SD: 1.71) for LLatThorFlex, respectively (S2 Table). Pearson correlation coefficients between all parameters in walk and trot varied between 0.406 and 0.842 (S3 and S4 Tables). Both in walk and in trot, Pearson correlation coefficients between the scores on GenMob and the scores on the eight other parameters showed correlations >0.5. Especially the scores on ThorFlex, LumbFlex, and LumbSacFlex were highly correlated with the GenMob scores (>0.63) (S3 and S4 Tables). In general, correlation scores of flexion and extension of the same part of the back, and scores of similar motions (flexion or extension) between adjacent parts were higher than with other parts (> 0.61) (S3 and S4 Tables).

**Fig 4:**
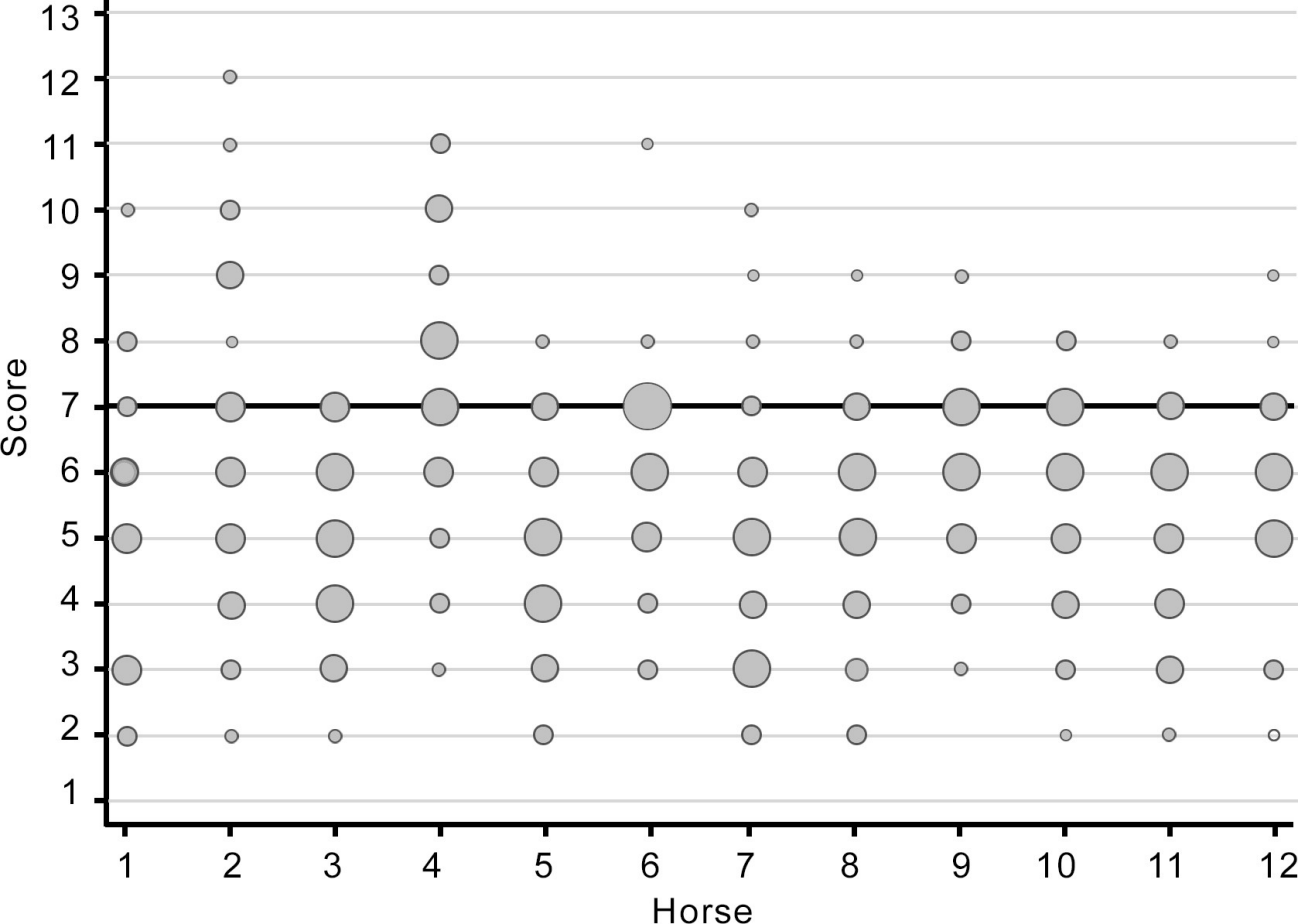
Score of 70 observers for GenMob (general mobility) in walk for each horse. There is a very large variation in the perception of the degree of back mobility, especially if and to what extent there is a decrease in mobility. This explains the low inter-rater agreement (ICC = 0.179). The size of dots illustrates the number of observations.

The ICCs of the nine parameters of the back of the horse both in walk and in trot were all beneath 0.2 (Table 2), indicating poor agreement between observers. The lowest ICC was found for the ThorFlex in walk (0.069) and the highest for GenMob in walk (0.179). This low inter-rater agreement is illustrated in Fig 4 for the parameter general mobility. Education, expertise or experience had no significant influence on the ICCs for back motion.

**Table 2:** Inter-rater reliability of scores and 95% confidence intervals of the 9 parameters at walk and trot expressed as ICC, n = 840 for both walk and trot.

*Parameter*	*ICC walk*	*ICC trot*
GenMob	0.179 (0.09–0.39)	0.114 (0.06–0.28)
ThorFlex	0.069 (0.03–0.19)	0.077 (0.03–0.21)
ThorExt	0.071 (0.03–0.20)	0.098 (0.05–0.25)
LumbFlex	0.075 (0.03–0.20)	0.075 (0.03–0.20)
LumbExt	0.109 (0.05–0.27)	0.082 (0.04–0.22)
LumbSacFlex	0.077 (0.03–0.21)	0.072 (0.03–0.20)
LumbSacExt	0.100 (0.05–0.25)	0.074 (0.03–0.20)
LLatThorFlex	0.145 (0.07–0.34)	0.090 (0.04–0.23)
RLatThorFlex	0.164 (0.08–0.37)	0.090 (0.04–0.23)

GenMob (general mobility), ThorFlex (thoracic flexion), ThorExt (thoracic extension), LumbFlex (lumbar flexion), LumbExt (lumbar extension), LumbSacFlex (lumbosacral flexion), LumbSacExt (lumbosacral extension), LLatThorFlex (left thoracolumbar latero-flexion), and RLatThorFlex (right thoracolumbar latero-flexion).

### Intra-rater reliability

**Fig 5:**
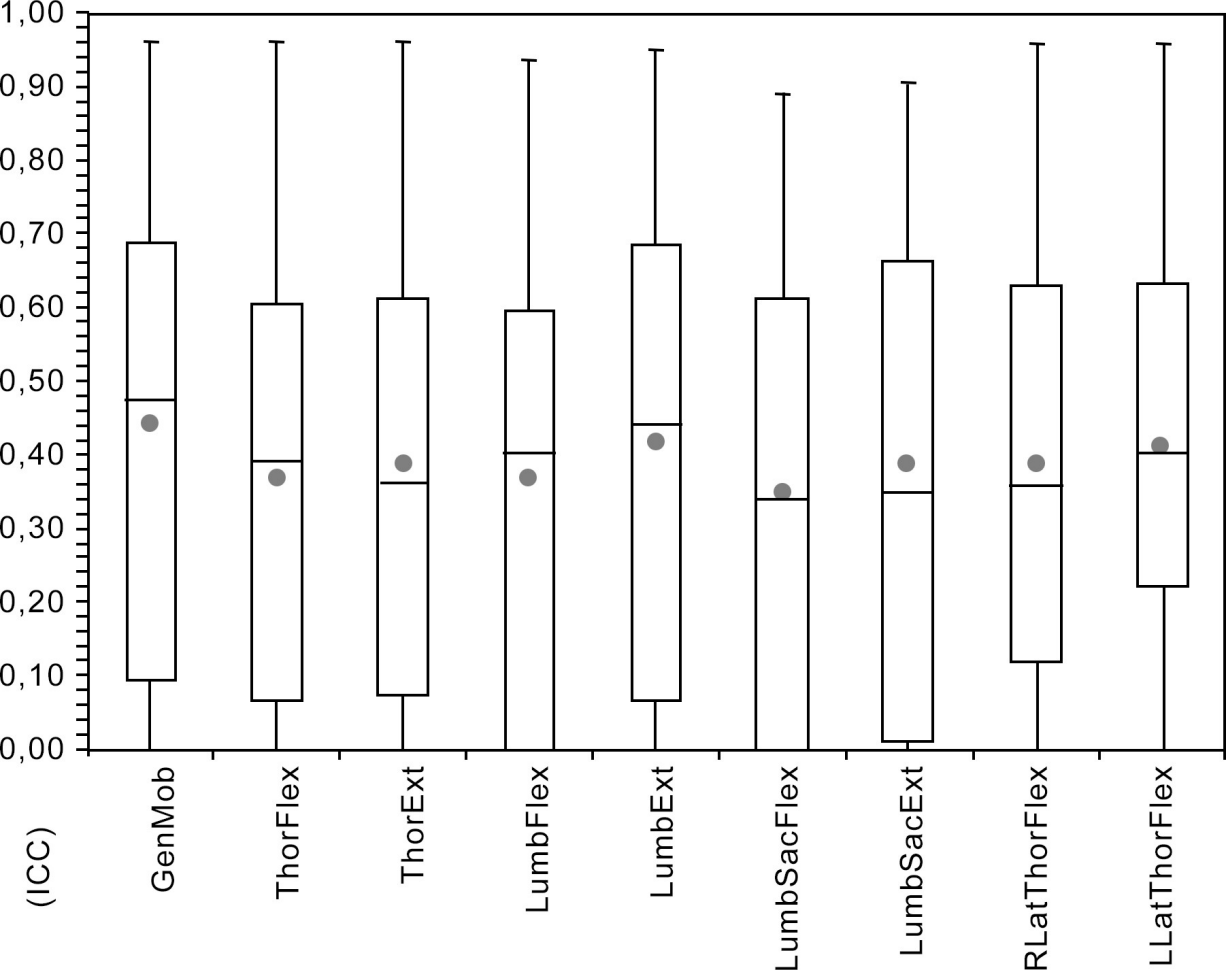
Box-Whisker plot (black dot: Mean, horizontal bar in box: Median) for intra-rater reliability (ICC) for general mobility (GenMob), thoracic flexion (ThorFlex), thoracic extension (ThorExt), lumbar flexion (LumbFlex), lumbar extension (LumbExt), lumbosacral flexion (LumbSacFlex), lumbosacral extension (LumbSacExt), left thoracolumbar latero-flexion (LLatThorFlex) and right thoracolumbar latero-flexion (RLatThorFlex).

The mean intra-rater reliability varied between 0.34 for LumbsacFlex and 0.44 for GenMob for the individual parameters, the intra-reliability varied between zero (poor), and 0.96 (excellent) (Fig 5). LumbFlex and LumbSacFlex had the lowest score, with 25% of observers scoring only random agreement for the repeated evaluation. The intra-rater reliability for LLatThorFlex was higher for physiotherapists compared to veterinarians (0.55 vs. 0.36; P = 0.04). Intra-rater reliability for RLatThorFlex was also numerically, but not significantly (P = 0.0736), higher for the physiotherapists compared to vets (0.51 vs. 0.34). The intra-rater reliability for LumbSacFlex was significantly higher for persons working fulltime with horses compared to those working part-time (0.45 vs. 0.26; P = 0.02). For all other parameters, the effect of education or expertise was not significant.

### Quantitative kinematic parameters: Comparison with visual scoring

The analysed kinematic data of all 12 horses in walk and trot is listed in S5 Table. For objective calculations of the whole back. general mobility was divided in whole back flexion/ extension and whole back lateral bending.

For each parameter, the average correlation between visual scoring and the gait analysis measurements was very low, overall, on average 0.02 for walk and -0.05 for trot, and varied between ~ -0.8 and 0.8 (Table 3). This indicates that on average there is no correlation between the visual subjective scoring and objective gait analysis measurements. Further, observers do not have solely positive or negative correlations with gait analysis measurements but might have a (high) negative correlation for one parameter and a (high) positive correlation for another. Also, for observers with a good intra-rater reliability (>0.8 for all parameters), the correlation of their visual scores with the objective measurements was low in walk as well as trot, varying between -0.008 and 0.14. This indicates that, even while they were consistent in evaluating the horses, there was no correlation with the objective measurements.

**Table 3:** Means (Mean), standard deviations (SD), minima (Min), maxima (Max) correlations between visual observations and objective measurements in walk and trot (abbreviations see Table 2).

		Walk				Trot			
Variable	N	Mean	SD^§^	Min	Max	Mean	SD^§^	Min	Max
GenMob (FE^†^)	70	0.25	0.32	-0.62	0.79	-0.05	0.26	-0.61	0.58
GenMob (LB^‡^)	70	0.15	0.32	-0.48	0.66	-0.03	0.28	-0.70	0.59
ThorFlex	70	0.07	0.29	-0.71	0.66	-0.14	0.26	-0.65	0.46
ThorExt	70	0.08	0.30	-0.57	0.77	-0.12	0.25	-0.60	0.49
LumbFlex	70	-0.17	0.30	-0.79	0.56	0.13	0.25	-0.56	0.63
LumbExt	70	-0.19	0.31	-0.82	0.66	0.06	0.29	-0.60	0.51
LumbSacFlex	70	0.04	0.28	-0.70	0.60	-0.03	0.26	-0.60	0.55
LumbSacExt	70	-0.09	0.29	-0.76	0.68	0.02	0.26	-0.55	0.61
RLatThorFlex	70	-0.04	0.28	-0.57	0.67	-0.24	0.31	-0.75	0.62
LLatThorFlex	70	0.05	0.29	-0.60	0.57	-0.09	0.33	-0.69	0.65

^†^ FE: flexion/ extension

^‡^ LB: lateral bending

^§^ SD: standard deviation.

## Discussion/Conclusions

The main findings in this study are that in scoring equine back motion there is poor inter-individual agreement between observers, irrespective of their experience or professional background, that intra-individual agreement is on average limited, but has a high variation between individuals, and that there was no correlation between the scoring of observers and the objective measurements, also in case the intra-rater agreement of the individual observer was high and again irrespective of degree of professional experience. It was expected that both a board-certified orthopedic surgeon or a sports medicine diplomate would be more experienced compared to other board-certified specialists, equine practitioners, generalists or students but this could not be demonstrated. This outcome leads to the overall conclusion that the 1^st^ and 3^rd^ hypothesis of the study have to be rejected. Where intra-rater agreement was better than inter-rater agreement and could even be excellent in individual cases, the average agreement was less than fair to good, meaning that the 2^nd^ hypothesis has to be rejected as well.

Because the clinical assessment serves as an important basis for decision making on treatment and in pre-purchase vetting of a horse, the apparently weak foundation for this assessment of back function urges caution in its interpretation. And it strongly supports the demand for more objective analysis of the functionality of the back of the horse, in analogy with the assessment of equine lameness [13, 14].

It can be argued that the study did not evaluate the overall rater agreement of back movement in horses, but the agreement on the scale that was designed. In fact, this is true, but not different from other studies in which scores on scales are compared, like the common lameness scores or scores on visual analogue scales, as are widely used in human medicine. Admittedly, people are more familiar with the common lameness scales than with a custom-made scale like the one used in this study, which we were forced to use as explained above. This we recognize as a limitation of this study. However, we thought it an inevitable one and not prohibitive. The only other option would have been to not carry out the study. The subject of the paper can only be addressed by use of some sort of a (semi-) quantitative scoring system. There are such systems for scoring lameness, but no such system exists for the assessment of back motion, which made the design of an own system unavoidable. Every practitioner does this in her or his own way, but the common goal of all of them is to assess whether back functionality is deviant from the physiological situation or not and whether it is affected enough to warrant some kind of intervention. For this reason, we included this consideration in the scoring system. We further made a relatively broad, detailed scale to avoid the observers feeling restricted in their choices. We want to emphasize that the system was used as a necessary tool only. It is not proposed for use in clinical practice, nor does the study pretend to validate the system in any way.

Poor inter- and intra-rater agreement has also been recognized in other studies on assessment of musculoskeletal system performance in both horses [8] and humans [15, 16]. In those latter studies not surprisingly low levels of agreement were scored on difficultly observable parameters like pelvic rotation and plantar flexion of the ankle in late stance phase in humans, with higher agreement on easily observable parameters, such as arm swing, knee extension and lateral flexion of the trunk [16]. Like in humans, equine back motion is complex and difficult to observe. One reason for this is the relatively small range of motion of thoracolumbar segments. Maximal intervertebral range of motion between T6 and T10 for flexion/extension during walk is 5.6° (+/- 2.0°) [5]. This amount of movement is objectively measurable but may be too difficult to appreciate with the human eye. Related to this is the fact that some asymmetries are undetectable for the human eye, even for experienced observers. A threshold of approximately 25% was found in a study for detection of asymmetry in movement of the equine tuber coxae movement [17]. In the current study, the observers may have benefitted from the markers on the horse, which “highlighted” the areas of interest. Still, even in the area with the largest range of motion, i.e. the lumbosacral area [1], intra-rater reliability was poor (0.34).

Another limitation of the study and a possible source of decreased agreement between and within observers was the use of videos [18, 19]. The videos were of high quality and had the advantage that they could be played multiple times, however, videos reduce depth and dimension perception compared to real time interpretation. On the other hand, pilot studies in human medicine have reported that rating frontal and sagittal videos was more reliable than evaluating live subjects [20]. In our situation, videos were used for logistical reasons, to make it possible to include a substantial number of participants and to standardize maximally the dynamic evaluation. Acknowledging that video assessment may negatively influence agreement, we expected small disagreements. However, the differences in agreement were quite substantial, making us look for other explanations.

Lack of agreement could also be caused by presenting horses only in walk and trot on a hard, straight line. Assessment on soft surface, lunging on both circles, canter, and a static examination were not included in this study and these additional observations might have improved agreement. However, although the use of circles has been proven to help in detecting front limb lameness more accurately [21], the agreement between experienced equine veterinarians evaluating lame horses on a straight line did not improve when circles were added to the examination [7]. Nevertheless, further research is indicated to verify if additionally lunging the horse would improve inter- and intra-rater reliability. Intuitively, adding canter to this study could also have been beneficial in the evaluation of flexion and extension of the lumbosacral area [4]. For other areas of the thoracolumbar spine, this is less probable, because in canter the back shows similar mobility as in walk [3–5].

A not unlikely reason for the poor correlation of the subjective scores with the quantitative data and the overdiagnosis of hypomobility is expectation bias. The observers did not have information on the clinical status of the horses and in a survey to assess back mobility it lies at hand to expect a certain number of back patients in which in general hypomobility will be the predominant sign. Expectation bias has been shown previously to affect the assessment of lameness in horses [22]. Another explanation for scoring ‘suspected pathology’ in a relatively high number of horses that in fact were deemed clinically sound is that, theoretically, forms of pathology may have existed that had remained undetected during the clinical exam. However, the large scale on which pathology was suspected and the huge variety of scores per individual horse make this theory unlikely. Another reason why this explanation is unlikely is that Hardeman et al. [10], when measuring range of motion and between measurement variation of spinal kinematics in 12 owner-sound horses in trot, found lower rather than higher ROM (Range Of Motion) for pelvis roll, pitch and yaw and for whole back flexion-extension and whole back lateral bending compared to the current study (S6 Table).

Average mobility scores were lower for trot than for walk. This outcome can probably be explained from the overall stiffer impression the equine back makes at trot compared to the walk and may hence point to insufficient recognition of the fact that this is insufficiently recognised as a physiological condition, as trot is well-documented to be a gait with less back motion compared to walk [5]. The better correlation of back mobility parameters between adjacent segments compared to segments further away from each other can be based on intuition and could, therefore, be expected as such.

It can be concluded that this study raises substantial concerns about the reliability of the current clinical practice of assessing back mobility in the horse on a straight line in walk and trot and hence about the value and validity of these assessments. A more objective way of characterizing back mobility in horses using computerized gait analysis systems, as has been developed for lameness exams, would almost certainly address these issues and research efforts into the development of such an approach are thus warranted.

## Supporting information

S1 Fig A screenshot of the survey. In this example the frontal and lateral video stills are displayed. All 9 items are scored and one box per item is ticked.
(TIFF)

S1 Table Scores on the 9 parameters of the horses back in walk. Means (Mean), standard deviations (SD), minima (Min), maxima (Max), medians (Med) and interquartile ranges (Q1 and Q3) of the scores on the 9 parameters of the horses back in walk over all horses. N = 840 (abbreviations see Table 2).
(DOCX)

S2 Table Scores on the 9 parameters of the horses back in trot. Means (Mean), standard deviations (SD), minima (Min), maxima (Max), medians (Med) and interquartile ranges (Q1 and Q3) of the scores on the 9 parameters of the horses back in trot over all horses. N = 840 (abbreviations see Table 2).
(DOCX)

S3 Table Pearson correlation coefficients between scores on the 9 parameters in walk. N = 840 (abbreviations see Table 2).
(DOCX)

S4 Table Pearson correlation coefficients between scores on the 9 parameters in trot. N = 840 (abbreviations see Table 2).
(DOCX)

S5 Table Calculated kinematic variables derived from gait analysis measurements. Median, 5% and 95% percentiles of all 12 horses on hard surface and over a straight line in walk and trot.
(DOCX)

S6 Table Main variables of back movement in trot. Comparison of pelvis roll, pitch, yaw and whole back flexion- extension and whole back lateral bending on hard straight surface in trot between horses in current study with those in Hardemans study [10].
(DOCX)
