# Supplementary figures and images for: Rater agreement for assessment of equine back mobility at walk and trot compared to quantitative gait analysis
Source: PLoS One. 2021 Jun 4;16(6):e0252536. doi: 10.1371/journal.pone.0252536 (PMC8177646; doi:10.1371/journal.pone.0252536)

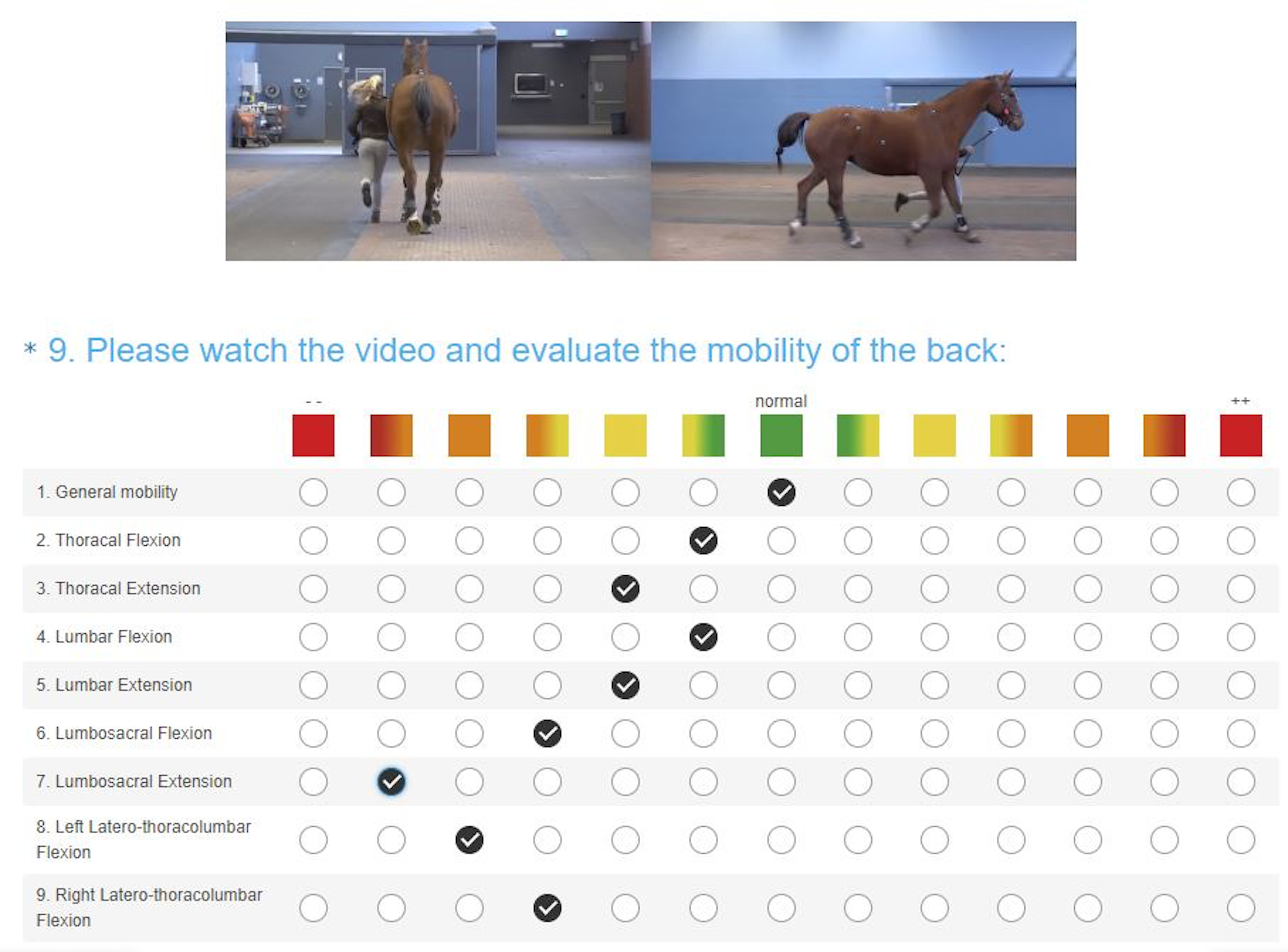

Supplement: S1 Fig — In this example the frontal and lateral video stills are displayed. All 9 items are scored and one box per item is ticked. (TIFF) [file pone.0252536.s001.tiff]
