# Supplementary material for: Rater agreement for assessment of equine back mobility at walk and trot compared to quantitative gait analysis
Source: PLoS One. 2021 Jun 4;16(6):e0252536. doi: 10.1371/journal.pone.0252536 (PMC8177646; doi:10.1371/journal.pone.0252536)
Supplement: S1 Table — Means (Mean), standard deviations (SD), minima (Min), maxima (Max), medians (Med) and interquartile ranges (Q1 and Q3) of the scores on the 9 parameters of the horses back in walk over all horses. N = 840 (abbreviations see Table 2). (DOCX) [file pone.0252536.s002.docx]

S1 Table: Scores on the 9 parameters of the horses back in walk.

Means (Mean), standard deviations (SD), minima (Min), maxima (Max), medians (Med) and interquartile ranges (Q1 and Q3) of the scores on the 9 parameters of the horses back in walk over all horses. N=840 (abbreviations see Table 2).

| Parameter | Mean | SD | Min | Max | Med | Q1 | Q3 |
| --- | --- | --- | --- | --- | --- | --- | --- |
| GenMob | 5.53 | 1.75 | 2 | 12 | 6 | 4 | 7 |
| ThorFlex | 5.39 | 1.69 | 1 | 11 | 5 | 4 | 7 |
| ThorExt | 5.69 | 1.77 | 1 | 11 | 6 | 4 | 7 |
| LumbFlex | 5.33 | 1.92 | 1 | 11 | 5 | 4 | 7 |
| LumbExt | 5.47 | 2.04 | 1 | 13 | 5 | 4 | 7 |
| LumbSacFlex | 5.29 | 2.02 | 1 | 13 | 5 | 4 | 7 |
| LumbSacExt | 5.56 | 2.15 | 1 | 13 | 6 | 4 | 7 |
| LLatThorFlex | 6.10 | 2.11 | 1 | 12 | 6 | 5 | 7 |
| RLatThorFlex | 6.02 | 2.09 | 1 | 12 | 6 | 5 | 7 |
