# Supplementary material for: Rater agreement for assessment of equine back mobility at walk and trot compared to quantitative gait analysis
Source: PLoS One. 2021 Jun 4;16(6):e0252536. doi: 10.1371/journal.pone.0252536 (PMC8177646; doi:10.1371/journal.pone.0252536)
Supplement: S2 Table — Means (Mean), standard deviations (SD), minima (Min), maxima (Max), medians (Med) and interquartile ranges (Q1 and Q3) of the scores on the 9 parameters of the horses back in trot over all horses. N = 840 (abbreviations see Table 2). (DOCX) [file pone.0252536.s003.docx]

S2 Table: Scores on the 9 parameters of the horses back in trot.

Means (Mean), standard deviations (SD), minima (Min), maxima (Max), medians (Med) and interquartile ranges (Q1 and Q3) of the scores on the 9 parameters of the horses back in trot over all horses. N=840 (abbreviations see Table 2).

| Parameter | Mean | SD | Min | Max | Med | Q1 | Q3 |
| --- | --- | --- | --- | --- | --- | --- | --- |
| GenMob | 4.96 | 1.55 | 1 | 10 | 5 | 4 | 6 |
| ThorFlex | 4.98 | 1.65 | 1 | 11 | 5 | 4 | 6 |
| ThorExt | 5.22 | 1.83 | 1 | 12 | 5 | 4 | 6.5 |
| LumbFlex | 4.84 | 1.85 | 1 | 11 | 5 | 3 | 6 |
| LumbExt | 4.94 | 1.96 | 1 | 12 | 5 | 3 | 6 |
| LumbSacFlex | 4.78 | 1.87 | 1 | 11 | 5 | 3 | 6 |
| LumbSacExt | 4.87 | 1.90 | 1 | 13 | 5 | 3 | 6 |
| LLatThorFlex | 5.24 | 1.71 | 1 | 11 | 5 | 4 | 7 |
| RLatThorFlex | 5.23 | 1.73 | 1 | 12 | 5 | 4 | 7 |
