# Supplementary material for: Rater agreement for assessment of equine back mobility at walk and trot compared to quantitative gait analysis
Source: PLoS One. 2021 Jun 4;16(6):e0252536. doi: 10.1371/journal.pone.0252536 (PMC8177646; doi:10.1371/journal.pone.0252536)
Supplement: S3 Table — N = 840 (abbreviations see Table 2). (DOCX) [file pone.0252536.s004.docx]

S3 Table: Pearson Correlation Coefficients between scores on the 9 parameters in walk. N=840 (abbreviations see Table 2).

| Pearson correlation coefficients. N = 840 | | | | | | | | | |
| --- | --- | --- | --- | --- | --- | --- | --- | --- | --- |
|  | Gen Mob | Thor Flex | Thor Ext | Lumb Flex | Lumb Ext | Lumb Sac Flex | Lumb Sac Ext | LLat Thor Flex | RLat Thor Flex |
| GenMob | 1 |  |  |  |  |  |  |  |  |
| ThorFlex | 0.665 | 1 |  |  |  |  |  |  |  |
| ThorExt | 0.524 | 0.708 | 1 |  |  |  |  |  |  |
| LumbFlex | 0.637 | 0.614 | 0.566 | 1 |  |  |  |  |  |
| LumbExt | 0.577 | 0.531 | 0.619 | 0.651 | 1 |  |  |  |  |
| LumbSacFlex | 0.662 | 0.554 | 0.537 | 0.801 | 0.589 | 1 |  |  |  |
| LumbSacExt | 0.556 | 0.478 | 0.578 | 0.609 | 0.776 | 0.703 | 1 |  |  |
| LLatThorFlex | 0.510 | 0.406 | 0.428 | 0.431 | 0.436 | 0.469 | 0.461 | 1 |  |
| RLatThorFlex | 0.574 | 0.454 | 0.474 | 0.478 | 0.438 | 0.499 | 0.479 | 0.709 | 1 |
