# Supplementary material for: Rater agreement for assessment of equine back mobility at walk and trot compared to quantitative gait analysis
Source: PLoS One. 2021 Jun 4;16(6):e0252536. doi: 10.1371/journal.pone.0252536 (PMC8177646; doi:10.1371/journal.pone.0252536)
Supplement: S4 Table — N = 840 (abbreviations see Table 2). (DOCX) [file pone.0252536.s005.docx]

S4 Table: Pearson Correlation Coefficients between scores on the 9 parameters in trot. N=840 (abbreviations see Table 2).

| Pearson correlation coefficients. N = 840 | | | | | | | | | |
| --- | --- | --- | --- | --- | --- | --- | --- | --- | --- |
|  | Gen Mob | Thor Flex | Thor Ext | Lumb Flex | Lumb Ext | Lumb Sac Flex | Lumb Sac Ext | LLat Thor Flex | RLat Thor Flex |
| GenMob | 1 |  |  |  |  |  |  |  |  |
| ThorFlex | 0.746 | 1 |  |  |  |  |  |  |  |
| ThorExt | 0.601 | 0.644 | 1 |  |  |  |  |  |  |
| LumbFlex | 0.683 | 0.736 | 0.521 | 1 |  |  |  |  |  |
| LumbExt | 0.568 | 0.483 | 0.760 | 0.568 | 1 |  |  |  |  |
| LumbSacFlex | 0.666 | 0.716 | 0.539 | 0.834 | 0.558 | 1 |  |  |  |
| LumbSacExt | 0.573 | 0.512 | 0.720 | 0.560 | 0.842 | 0.613 | 1 |  |  |
| LLatThorFlex | 0.624 | 0.601 | 0.598 | 0.569 | 0.604 | 0.566 | 0.614 | 1 |  |
| RLatThorFlex | 0.575 | 0.547 | 0.574 | 0.535 | 0.592 | 0.530 | 0.615 | 0.777 | 1 |
