# Supplementary material for: Rater agreement for assessment of equine back mobility at walk and trot compared to quantitative gait analysis
Source: PLoS One. 2021 Jun 4;16(6):e0252536. doi: 10.1371/journal.pone.0252536 (PMC8177646; doi:10.1371/journal.pone.0252536)
Supplement: S5 Table — Median, 5% and 95% percentiles of all 12 horses on hard surface and over a straight line in walk and trot. (DOCX) [file pone.0252536.s006.docx]

S5 Table. Calculated kinematic variables derived from gait analysis measurements.

Median, 5% and 95% percentiles of all 12 horses on hard surface and over a straight line in walk and trot.

| Variable | Unit | Hard Straight | | | | | |
| --- | --- | --- | --- | --- | --- | --- | --- |
|  |  | Walk | | | Trot | | |
|  |  | 5% | median | 95% | 5% | median | 95% |
| Stride duration | sec | 1.19 | 1.24 | 1.29 | 0.73 | 0.76 | 0.79 |
| Stride frequency | Hz | 0.76 | 0.81 | 0.85 | 1.26 | 1.32 | 1.38 |
| Speed | m/s | 1.42 | 1.51 | 1.61 | 2.87 | 3.19 | 3.44 |
| Head ROM | mm | 119.37 | 146.33 | 175.42 | 60.84 | 76.97 | 95.47 |
| Withers ROM | mm | 36.64 | 42.42 | 47.81 | 82.95 | 89.91 | 97.65 |
| Sacrum ROM | mm | 70.97 | 78.89 | 87.03 | 84.53 | 93.05 | 103.08 |
| Pelvis roll (AR) | deg | 9.70 | 11.36 | 13.08 | 5.85 | 7.35 | 9.50 |
| Pelvis pitch (FE) | deg | 7.31 | 8.37 | 10.70 | 8.10 | 8.91 | 10.86 |
| Pelvis yaw (LB) | deg | 8.28 | 9.52 | 11.24 | 3.57 | 4.21 | 6.45 |
| Body tracking | deg | -1.38 | 0.31 | 1.98 | -1.49 | 0.56 | 2.26 |
| Head swivel | deg | -6.49 | 0.50 | 8.05 | -3.33 | 2.14 | 9.57 |
| Whole back FE | deg | 3.38 | 4.05 | 4.90 | 4.60 | 5.16 | 6.24 |
| Whole back LB | deg | 10.85 | 12.24 | 13.56 | 6.24 | 7.02 | 8.27 |
| T12 FE | deg | 4.21 | 4.95 | 6.44 | 4.03 | 4.68 | 5.61 |
| T12 LB | deg | 6.64 | 8.01 | 9.63 | 6.50 | 7.79 | 9.49 |
| T15 FE | deg | 1.84 | 2.65 | 4.78 | 1.91 | 2.51 | 3.44 |
| T15 LB | deg | 3.90 | 4.82 | 6.47 | 3.61 | 4.69 | 6.14 |
| T18 FE | deg | 1.92 | 2.81 | 4.23 | 1.71 | 2.39 | 3.71 |
| T18 LB | deg | 4.97 | 6.03 | 7.50 | 3.34 | 4.10 | 5.55 |
| L3 FE | deg | 2.24 | 2.82 | 4.04 | 2.20 | 2.75 | 3.77 |
| L3 LB | deg | 5.00 | 5.79 | 6.95 | 5.11 | 6.24 | 7.33 |
| L5 FE | deg | 3.47 | 4.32 | 5.57 | 2.76 | 3.45 | 4.70 |
| L5 LB | deg | 1.99 | 2.90 | 4.96 | 3.43 | 4.24 | 5.99 |
| TS FE | deg | 3.10 | 3.78 | 5.01 | 2.87 | 3.71 | 4.95 |
| TS LB | deg | 3.41 | 4.11 | 5.93 | 3.57 | 4.30 | 5.89 |

ROM: range of motion in the vertical plane, AR: axial rotation, FE: flexion/ extension, LB: lateral bending, T: thoracic, L: lumbar, TS: Tuber Sacrale, deg: degree.
