# Supplementary material for: Rater agreement for assessment of equine back mobility at walk and trot compared to quantitative gait analysis
Source: PLoS One. 2021 Jun 4;16(6):e0252536. doi: 10.1371/journal.pone.0252536 (PMC8177646; doi:10.1371/journal.pone.0252536)
Supplement: S6 Table — Comparison of pelvis roll, pitch, yaw and whole back flexion- extension and whole back lateral bending on hard straight surface in trot between horses in current study with those in Hardemans study [10]. (DOCX) [file pone.0252536.s007.docx]

S6 Table: Main variables of back movement in trot.

Comparison of pelvis roll, pitch, yaw and whole back flexion- extension and whole back lateral bending on hard straight surface in trot between horses in current study with those in Hardemans study [10].

| Variable | Unit | Hardeman [10] | | | Current study | | |
| --- | --- | --- | --- | --- | --- | --- | --- |
|  |  | 5% | median | 95% | 5% | median | 95% |
| Pelvis roll (AR^†^) | deg | 5.84 | 8.51 | 9.08 | 5.85 | 7.35 | 9.50 |
| Pelvis pitch (FE^‡^) | deg | 4.92 | 6.98 | 8.16 | 8.10 | 8.91 | 10.86 |
| Pelvis yaw (LB^§^) | deg | 3.12 | 3.95 | 4.94 | 3.57 | 4.21 | 6.45 |
| Whole back FE | deg | 3.98 | 4.91 | 5.61 | 4.60 | 5.16 | 6.24 |
| Whole back LB | deg | 4.96 | 6.46 | 8.08 | 6.24 | 7.02 | 8.27 |

^†^ AR: axial rotation, ^‡^ FE: flexion/ extension, ^§^ LB: lateral bending.
